# Supplementary material for: User Experience during an Immersive Virtual Reality-Based Cognitive Task: A Comparison between Estonian and Italian Older Adults with MCI
Source: Sensors (Basel). 2022 Oct 27;22(21):8249. doi: 10.3390/s22218249 (PMC9657959; doi:10.3390/s22218249)
Supplement: Supplementary file 1 [file sensors-22-08249-s001.zip › sensors-1972897-supplementary.pdf]

Table S1: Correlations coefficients between SSQ and ITC-SOPI side effects subscale pre and post-experience in the Virtual Supermarket;

	**SSQ-N pre**	**SSQ-O pre**	**SSQ-D pre**	**SSQ-TS pre**	**SSQ-N post**	**SSQ-O post**	**SSQ-D post**	**SSQ-TS post**	**SE post**
**SSQ-N pre**									
**SSQ-O pre**	$.836^{\S}$								
**SSQ-D pre**	$.542^{*}$	$.687^{\S}$							
**SSQ-TS pre**	$.841^{\S}$	$.961^{\S}$	$.751^{\S}$						
**SSQ-N post**	.357	.456	.056	.437					
**SSQ-O post**	$.644^{\S}$	$.603^{*}$	$.570^{*}$	$.672^{\S}$	$.585^{*}$				
**SSQ-D post**	.265	.414	$.742^{\S}$	.465	.301	$.793^{\S}$			
**SSQ-TS post**	$.536^{*}$	$.608^{*}$	$.561^{*}$	$.666^{\S}$	$.695^{\S}$	$.960^{\S}$	$.821^{\S}$		
**SE post**	$.569^{*}$	$.745^{\S}$	$.709^{\S}$	$.780^{\S}$	$.662^{\S}$	$.855^{\S}$	$.785^{\S}$	$.918^{\S}$	

N: nausea,
O: oculomotor disturbances,
D: disorientation,
TS: total score
SE: side effects
$^{*}p < 0.05$, $^{\S}p < 0.01$.

Table S2: Correlations coefficients between ITC-SOPI subscales.

	**SP**	**ENG**	**N**	**SE**
**SP**	-			
**ENG**	.835§			
**N**	.890§	.752§		
**SE**	-.240	-.140	.019	-

SP: spatial presence,
ENG: engagement,
N: naturalness,
SE: side effects,
$^{\S}p < 0.01$.

Table S3: Correlations coefficients between TAM3 subscales and SSQ subscales before the experience in the Virtual Supermarket.

	**BI pre**	**BI**	**PEOU**	**CANX**	**ENJ**	**SSQ-N pre**	**SSQ-O pre**	**SSQ-D pre**	**SSQ-TS pre**
**BI pre**	-								
**BI**	.698§								
**PEOU**	.434	.552*							
**CANX**	-.148	-.464	-.503						
**ENJ**	.653§	.732§	.841§	-.510					
**SSQ-N pre**	-.254	.114	-.190	-.299	-.111				
**SSQ-O pre**	-.427	-.127	-.451	-.256	-.434	.743§			
**SSQ-D pre**	-.349	-.180	-.530*	-.042	-.585*	.323	.789§		
**SSQ-TS pre**	-.396	-.060	-.419	-.262	-.390	.850§	.979§	.743§	-

BI: behavioral intention,
PEOU: perceived ease-of-use,
CANX: computer anxiety,
ENJ: enjoyment,
N: nausea,
O: oculomotor disturbances,
D: disorientation,
TS: total score,
$^{*}p < 0.05$, $^{\S}p < 0.01$.

Table S4: Correlations coefficients between TAM3 subscales and SSQ subscales after the experience in the Virtual Supermarket.

	**BI pre**	**BI**	**PEOU**	**CANX**	**ENJ**	**SSQ-N post**	**SSQ-O post**	**SSQ-D post**	**SSQ-TS post**
**BI pre**	-								
**BI**	$.698^{§}$								
**PEOU**	.434	$.552^{*}$							
**CANX**	-.148	-.464	-.503						
**ENJ**	$.653^{§}$	$.732^{§}$	$.841^{§}$	-.510					
**SSQ-N post**	-.385	-.026	-.241	.043	-.056				
**SSQ-O post**	-.205	-.082	-.506	.189	-.301	$.570^{*}$			
**SSQ-D post**	-.358	-.338	$-.666^{§}$	.434	-.514	.513	$.647^{§}$		
**SSQ-TS post**	-.357	-.156	$-.545^{*}$	.245	-.329	$.813^{§}$	$.901^{§}$	$.821^{§}$	-

BI: behavioral intention,
PEOU: perceived ease-of-use,
CANX: computer anxiety,
ENJ: enjoyment,
N: nausea,
O: oculomotor disturbances,
D: disorientation,
TS: total score,
$^{*}p < 0.05$, $^{§}p < 0.01$.

Table S5: Correlations coefficients between TAM3 and ITC-SOPI subscales.

	**BI pre**	**BI**	**PEOU**	**CANX**	**ENJ**	**SP**	**ENG**	**N**	**SE**
**BI pre**	-								
**BI**	.698§								
**PEOU**	.434	.552*							
**CANX**	-.148	-.464	-.503						
**ENJ**	.653§	.732§	.841§	-.510					
**SP**	.528*	.481	.219	-.046	.42				
**ENG**	.449	.572*	.373	-.340	.576*	.787§			
**N**	.331	.443	.053	.093	.198	.879§	.657§		
**SE**	-.523*	-.297	-.646§	.202	-.521*	-.125	-.242	.081	-

BI: behavioral intention,
PEOU: perceived ease-of-use,
CANX: computer anxiety,
ENJ: enjoyment,
SP: spatial presence,
ENG: engagement,
N: naturalness,
SE: side effects,
$^{*}p < 0.05$, $^{§}p < 0.01$.
